# Long-term efficacy and safety of osilodrostat in Cushing’s disease: final results from a Phase II study with an optional extension phase (LINC 2)

**DOI:** 10.1007/s11102-022-01280-6

**Published:** 2022-10-11

**Authors:** Maria Fleseriu, Beverly M. K. Biller, Jérôme Bertherat, Jacques Young, Betul Hatipoglu, Giorgio Arnaldi, Paul O’Connell, Miguel Izquierdo, Alberto M. Pedroncelli, Rosario Pivonello

**Affiliations:** 1 Pituitary Center, Departments of Medicine and Neurological Surgery, Oregon Health & Science University, Portland, OR USA; 2 Neuroendocrine and Pituitary Tumor Clinical Center, Massachusetts General Hospital, Boston, MA USA; 3 Department of Endocrinology, Centre de Référence des Maladies Rares de la Surrénale, Hôpital Cochin, AP-HP, Université de Paris, Paris, France; 4 Department of Endocrinology, Assistance Publique Hôpitaux de Paris, Bicêtre Hospital, Paris Saclay University, Le Kremlin-Bicêtre, France; 5 CWRU School of Medicine, University Hospital Cleveland Medical Center, Cleveland, OH USA; 6 Division of Endocrinology, Department of Clinical and Molecular Sciences, Polytechnic University of Marche, Ancona, Italy; 7 Novartis Ireland Limited, Dublin, Ireland; 8 Novartis Pharma AG, Basel, Switzerland; 9 Recordati AG, Basel, Switzerland; 10 Dipartimento di Medicina Clinica e Chirurgia, Sezione di Endocrinologia, Università Federico II di Napoli, Naples, Italy

**Keywords:** Cushing’s disease, Cushing’s syndrome, Osilodrostat, Cortisol, Hypercortisolism, Steroidogenesis inhibitor

## Abstract

**Background:**

Many patients with Cushing’s disease (CD) require long-term medical therapy to control their hypercortisolism. In the core phase of a Phase II study (LINC 2; NCT01331239), osilodrostat normalized mean urinary free cortisol (mUFC) in 78.9% of patients with CD. Here, we report long-term efficacy and safety data for osilodrostat following completion of an optional extension to LINC 2.

**Methods:**

Adult patients with CD were enrolled in a 22-week prospective Phase II study. Patients with mUFC ≤ upper limit of normal (ULN) or receiving clinical benefit at week 22 could enter the optional extension. The proportion of complete (mUFC ≤ ULN) or partial (mUFC > ULN but ≥ 50% decrease from baseline) mUFC responders was assessed over time.

**Results:**

Sixteen of 19 enrolled patients entered the extension. Median (range) osilodrostat exposure from baseline to study end was 5.4 years (0.04–6.7); median (range) average dose was 10.6 mg/day (1.1–47.9). Overall response rate (complete and partial mUFC responders) was consistently ≥ 50%. Sustained control of most cardiovascular-related parameters was observed during the extension. The long-term safety profile was consistent with that reported during the core phase. Testosterone levels (females) decreased towards baseline levels during long-term follow-up, with no new or worsening cases of hirsutism during the extension.

**Conclusions:**

In the longest prospective study of a steroidogenesis inhibitor to date, osilodrostat provided sustained reductions in mUFC for up to 6.7 years of treatment, with no new safety signals emerging during the extension. These findings support osilodrostat as an effective long-term treatment for patients with CD.

**Supplementary Information:**

The online version contains supplementary material available at 10.1007/s11102-022-01280-6.

## Introduction

Cushing’s disease, the most common form of endogenous Cushing’s syndrome, is a rare, debilitating disorder that is characterized by increased secretion of adrenocorticotropic hormone (ACTH) by a pituitary tumor and overproduction of cortisol by the adrenal glands [1, 2]. Chronic exposure to excess cortisol levels is associated with a broad spectrum of morbidity, including metabolic, cardiovascular and psychiatric disorders, that negatively impacts quality of life (QoL) and mortality [1–3]. Therefore, normalizing cortisol levels is a key treatment goal in Cushing’s disease to alleviate comorbidities, improve QoL and reduce mortality [1, 4].

Transsphenoidal pituitary surgery is the first-line treatment option for most patients with Cushing’s disease [1, 5]. However, approximately one-third of patients require further treatment such as repeat pituitary surgery, pituitary radiotherapy, bilateral adrenalectomy or medical therapy to control cortisol levels because of a failure to respond to surgery or disease recurrence [1, 4, 6]. For these patients and for those who refuse or are not suitable candidates for surgery, medical therapies represent an important therapeutic option. Therefore, it is essential to understand the long-term efficacy and safety profile of any medical therapy used to treat this chronic condition [1, 7, 8].

Osilodrostat is a potent oral inhibitor of 11β-hydroxylase, the enzyme that catalyzes the final step of cortisol synthesis in the adrenal glands [9, 10]. It is approved in the EU, Switzerland and Japan for the treatment of adult patients with endogenous Cushing’s syndrome for whom surgery is not an option or has not been curative [11–13], and in the USA for the treatment of adult patients with Cushing’s disease [14].

Findings from the core phase of a Phase II, open-label, prospective study of osilodrostat in patients with Cushing’s disease (LINC 2; ClinicalTrials.gov identifier: NCT01331239) showed that 78.9% (n/N = 15/19) of patients achieved normal mean 24-h urinary free cortisol (mUFC) at the end of the core phase (week 22), and that osilodrostat had a favorable safety profile [15]. This manuscript reports the final long-term efficacy and safety data from this Phase II study following completion of an optional, long-term extension phase.

## Methods

### Patients

As reported previously, patients aged 18–75 years with a confirmed diagnosis of persistent, recurrent or de novo (if non-surgical candidates) Cushing’s disease were enrolled in the LINC 2 Phase II study assessing the efficacy and safety of osilodrostat [15]. Active Cushing’s disease was confirmed at screening according to the following criteria: mUFC level above the upper limit of normal (ULN; 138 nmol/24 h or 50 µg/24 h) for patients who had completed a previous proof-of-concept study (LINC 1) [10] and a 14-day washout period after the study had ended, *or* > 1.5 × ULN for newly enrolled patients; morning plasma ACTH level above the lower limit of normal; and confirmation of a pituitary source of excess ACTH. Key exclusion criteria included a history of hypersensitivity to other steroidogenesis inhibitors, presence of high risk of optic chiasm compression, renal impairment, poorly controlled diabetes (glycated hemoglobin [HbA_1c_] > 9%), and risk factors for QTc prolongation or torsades de pointes.

### Study design

Patients initiated open-label osilodrostat at a dose of 2 mg twice daily (bid) during the 22-week core study, as previously described [15]. The osilodrostat dose was titrated within the range of 2–50 mg bid based on individual efficacy (if mUFC > ULN) and tolerability. One patient received osilodrostat 50 mg bid during the core phase. Following a post hoc analysis of a study of healthy volunteers that showed evidence of prolongation of corrected QT interval by Fridericia (QTcF) in some participants at single higher doses of osilodrostat (100 mg and 200 mg), a protocol amendment was implemented during the core phase that reduced the maximum permitted dose from 50 mg bid to 30 mg bid.

At week 22, patients with mUFC ≤ ULN or who were considered by the investigator to be receiving clinical benefit from osilodrostat were eligible for inclusion in an additional 48-week, optional extension phase (extension period 1) and continued with the same osilodrostat dose that they were receiving at week 22. Following a further 48 weeks of treatment (week 70; end of extension period 1), patients who were considered by the study investigator to be achieving clinical benefit from continued treatment with osilodrostat had the option of entering an additional, open-ended period (week 70 onwards; extension period 2). Dose adjustments were permitted during extension periods 1 and 2 based on individual efficacy and tolerability (maximum dose 30 mg bid). The study ended when a separate rollover study became available for patients receiving clinical benefit from osilodrostat or by December 31, 2019, whichever was earlier. A study completer was defined as a patient who completed their end-of-treatment visit in extension period 2 and transitioned to the rollover study.

The study was conducted in accordance with the Declaration of Helsinki, with an independent ethics committee/institutional review board at each site approving the study protocol; patients provided written informed consent to participate. The trial is registered at ClinicalTrials.gov (NCT01331239).

### Outcomes and assessments

During extension periods 1 and 2, patients attended a scheduled visit monthly for the first 6 months, every 3 months for the next 12 months, and every 6 months thereafter. The last patient’s last visit was on October 22, 2019.

The key objective of the long-term extension study was to assess the long-term efficacy and safety of osilodrostat beyond 22 weeks of treatment. The proportion of controlled (mUFC ≤ ULN) and partially controlled (mUFC > ULN but with ≥ 50% reduction from baseline) mUFC responders was assessed over time; the overall response rate was calculated as the sum of controlled and partially controlled mUFC responders. Patients who discontinued the study for any reason were classed as non-responders after discontinuation up to the furthest study visit that they could have completed at the time of data cut-off. Baseline mUFC measurements were calculated based on the mean of three 24-h urine samples collected within 14 days before the first dose. During treatment, mUFC was calculated from at least two 24-h urine specimens collected within 7 days of the relevant time point. mUFC was measured at a central laboratory by liquid chromatography-tandem mass spectrometry (Quest Diagnostics, Valencia, CA, USA).

Other biochemical parameters assessed at baseline and over time, as described previously [15], included serum morning cortisol, morning and late-night salivary cortisol, plasma ACTH, 11-deoxycortisol, 11-deoxycorticosterone, plasma aldosterone, plasma renin, testosterone, and HbA_1c_. Mean changes from baseline were calculated at week 22 (end of core phase), week 70 (end of extension period 1) and each patient’s last observed value (LOV).

Safety was assessed from baseline to study end by monitoring adverse events (AEs) according to Common Terminology Criteria for Adverse Events (version 4.03). AEs of special interest included events related to accumulation of adrenal hormone precursors, hypocortisolism, arrhythmogenic potential, and QT prolongation. Other safety assessments were related to laboratory evaluations (hematology, blood biochemistry and urinalysis). Concomitant medications (excluding drugs for the treatment of Cushing’s disease and drugs known to prolong the QT interval) were permitted at the discretion of the investigator. Glucocorticoids were permitted only for the short-term management of adrenal insufficiency.

### Statistical methods

Analyses were conducted following completion of the last patient’s last visit in the extension phase. Analysis of efficacy and safety was conducted for all enrolled patients who received at least one dose of osilodrostat. The proportion of mUFC responders over time was summarized using point estimates; patients who discontinued from the study were classed as mUFC non-responders at subsequent visits (up to the last evaluable assessment that they could have completed). The proportion of mUFC responders over time was also assessed using the LOV for each patient prior to study discontinuation or completion. Loss of mUFC response was defined as mUFC > ULN on ≥ 2 consecutive visits at the highest tolerated dose of osilodrostat after previously attaining mUFC normalization. Changes in biochemical, laboratory and cardiovascular-related parameters from baseline were analyzed descriptively. Data are presented using frequencies and percentages, mean and standard deviation (SD), or median and range, unless otherwise specified.

## Results

### Patient disposition

**Fig. 1:**
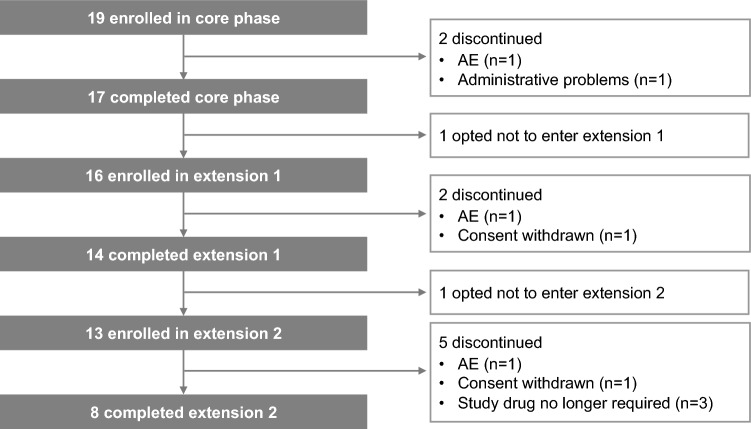
Patient disposition in the core and extension phases

Of 19 patients who were enrolled in the 22-week core phase (female, 73.7%; mean [SD] age, 36.8 years [8.4]; Table 1), 16 patients continued to receive osilodrostat during the extension phase (Fig. 1). Median (range) exposure to osilodrostat from core baseline to study end for all enrolled patients was 5.4 years (0.04–6.7). Median average osilodrostat dose from baseline to the end of the extension was 10.6 mg/day (range 1.1–47.9, interquartile range [IQR] 5.7–16.7) and median average dose with the longest duration was 10.0 mg/day (range 1.0–60.0, IQR 4.0–20.0). Fourteen (87.5%) and eight (50%) patients completed extension periods 1 and 2, respectively. Seven patients discontinued during extension periods 1 and 2, and one patient who completed extension period 1 (week 70) opted not to enter extension period 2 (Fig. 1).

**Table 1:** Patient baseline characteristics

Demographic variable	All patients, n = 19
Age, years	
Mean (SD)	36.8 (8.4)
Median (range)	36.0 (25–52)
Sex, n (%)	
Female	14 (73.7)
Male	5 (26.3)
Race, n (%)	
White	15 (78.9)
Black or African American	3 (15.8)
Asian	1 (5.3)
Previous pituitary surgery, n (%)	
Yes	17 (89.5)
No	2 (10.5)
Mean baseline UFC, x ULN* (SD)	9.9 (19.8)

Patient baseline characteristics have been published previously [15]

*ULN = 138 nmol/24 h

Over half of enrolled patients received osilodrostat for more than 4.5 years, and three patients remained on osilodrostat treatment for 6.5–6.7 years.

## Efficacy

### Changes in cortisol levels during long-term osilodrostat treatment

**Fig. 2:**
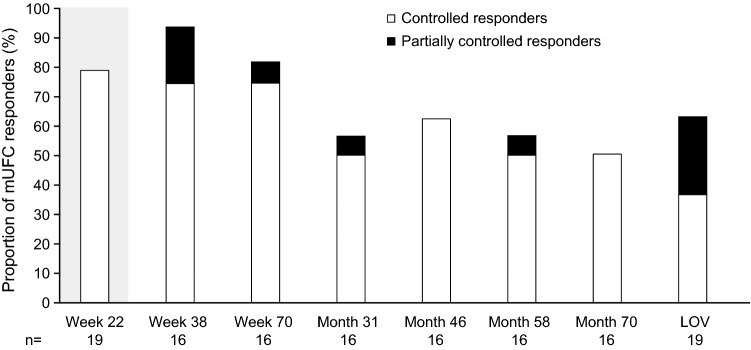
Proportion of mUFC responders over time*. Data highlighted by the shaded box have been published previously. 78.9% of enrolled patients (n = 15/19) were classified as overall mUFC responders at the end of the core phase (week 22): all of these patients were complete responders [15]. For patients who discontinued the study, their LOV was taken prior to study discontinuation, and they were not automatically classified as being non-responders at LOV. The two patients who discontinued during the core phase were classed as non-responders. *Patients who discontinued the study at any time were classed as non-responders at subsequent visits (up to the furthest study visit that they could have completed at the time of data cut-off)

Of the 16 patients who entered the extension phase, 81.3% (13/16) were overall mUFC responders at week 70: 75% (n = 12/16) were complete responders and 6.3% (n = 1/16) were partial responders. Overall, the mUFC response rate for all 16 patients enrolled in the extension study remained between 50 and 88% up to month 70 of the extension phase. Of 19 patients enrolled in the study, 63.2% (n = 12/19) of patients were complete (36.8% [n = 7/19]) or partial (26.3% [n = 5/19]) mUFC responders at their LOV (Fig. 2).

**Fig. 3:**
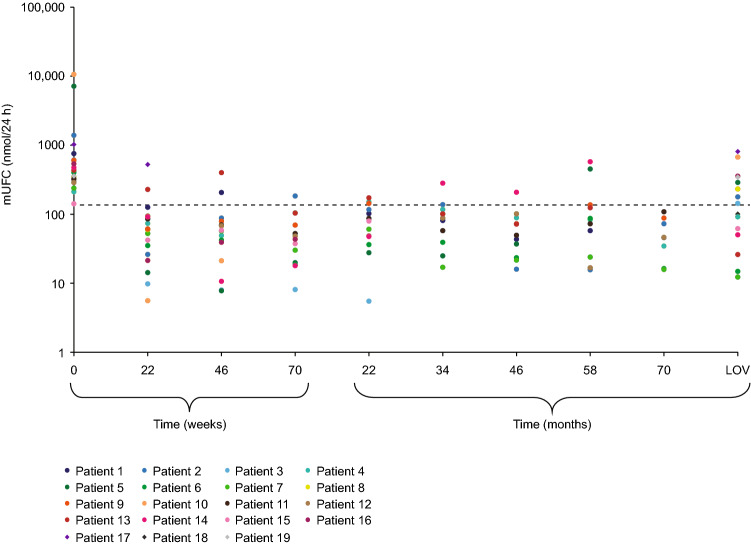
Individual patient mUFC data. Dashed line represents ULN (138 nmol/L)

The rapid reduction in mean mUFC observed during the core phase was maintained throughout the extension, with some patients remaining on osilodrostat treatment for up to 6.7 years. Mean (standard error [SE]) mUFC levels decreased from 9.9 × ULN (19.8) at study baseline to ≤ ULN at week 4 and remained within the normal range at most time points during the extension. UFC was < ULN in seven patients at LOV (Fig. 3).

Two patients met the study criteria for loss of mUFC response (mUFC > ULN on ≥ 2 consecutive visits at the highest tolerated osilodrostat dose after previously attaining mUFC normalization) during the extension phase. One patient lost control of mUFC after a reduction in osilodrostat dose because of an AE. The second patient had a loss of mUFC response at the highest tolerated osilodrostat dose at that time point of 7 mg/day, but they subsequently regained and sustained control of mUFC levels following a dose increase from 7 to 10 mg/day. Previously, the patient was on 10 mg and had a dose reduction to 7 mg/day; therefore, their highest tolerated dose at the time of the event was 7 mg/day.

Sustained reductions in mean mUFC levels with long-term osilodrostat treatment were accompanied by reductions in other cortisol parameters. Mean morning serum cortisol and morning salivary cortisol levels decreased to within the normal range during the core study and remained ≤ ULN during the extension phase. Individual patient data are presented in Supplementary Fig. 1a and b. Similarly, reductions in mean late-night salivary cortisol levels were maintained during long-term treatment. Individual patient data are presented in Supplementary Fig. 1c.

### Changes in cardiovascular-related parameters

**Fig. 4:**
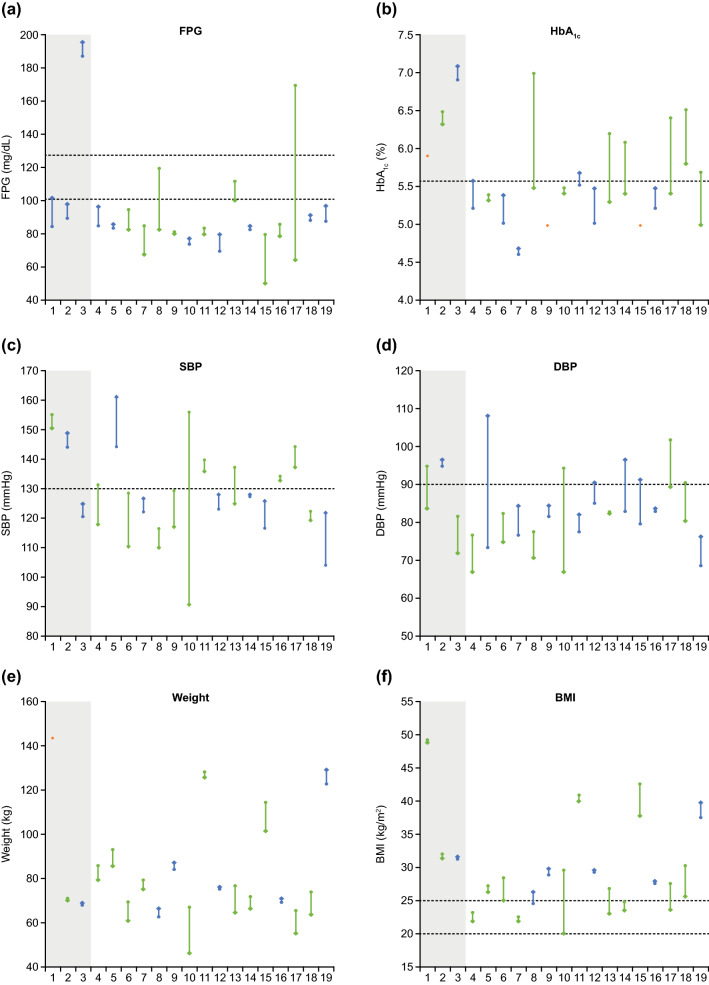
Individual patient data for **a** FPG, **b** HbA_1c_, **c** SBP, **d** DBP, **e **weight and **f** BMI. Patient 12 did not have a baseline value for HbA_1c_

Numerical decreases were seen in most cardiovascular-related parameters during the extension phase. At core baseline, 15/19 patients had FPG < 100 mg/dL; 16/19 patients had FPG < 100 mg/dL at their LOV. At baseline, 10/19 and 14/19 patients had SBP < 130 mmHg and DBP < 90 mmHg, respectively; at LOV, this was 13/19 and 13/19 patients, respectively. Overall, three patients who had SBP ≤ 130 mmHg and DBP ≤ 90 mmHg at baseline experienced a shift in DBP to > 90 mmHg at LOV (SBP remained ≤ 130 mmHg). Weight decreased from core baseline to LOV in 12/19 patients; BMI was within the normal range (18.5–24.9 kg/m^2^) in 3/19 patients at baseline and 7/19 patients at LOV (Fig. 4a–e). Mean HbA_1c_ levels were largely unchanged during the extension phase; 10/18 patients had HbA_1c_ < 5.6% at baseline, with 13/19 patients having HbA_1c_ < 5.6% at LOV (Fig. 4f). There were no clear changes in blood lipid levels (cholesterol, low-density lipoprotein [LDL] cholesterol, high-density lipoprotein [HDL] cholesterol, triglycerides) from baseline to LOV (Supplementary Fig. 2a–d).

### Changes in mean hormone levels

**Fig. 5:**
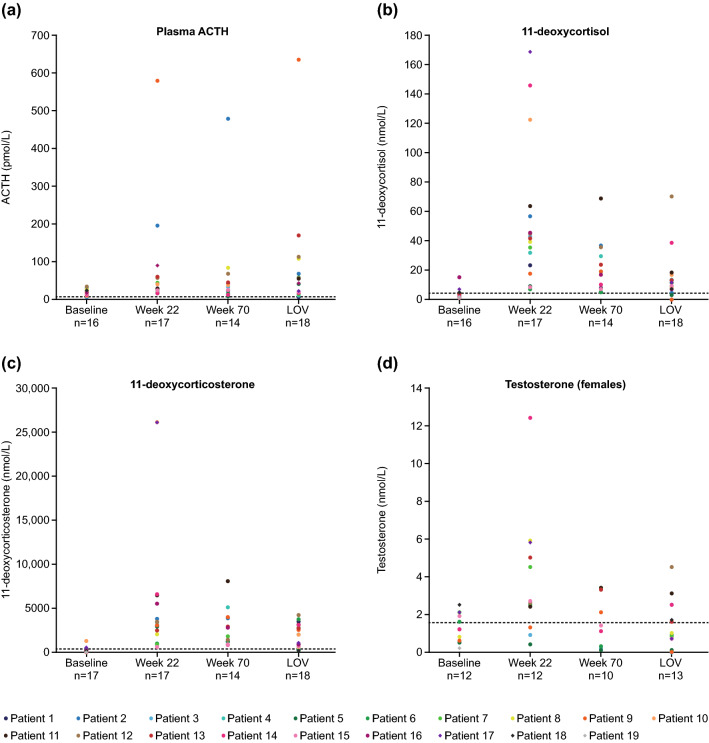
Individual patient data for **a** plasma ACTH, **b** 11-deoxycortisol, **c** 11-deoxycorticosterone and **d** testosterone (female) levels. Dashed lines represent ULN (plasma ACTH 9.2 pmol/L; 11-deoxycortisol 3.92 nmol/L; 11-deoxycorticosterone 3.95 nmol/L; testosterone [females] 1.6 nmol/L)

Plasma ACTH levels remained stable during the extension phase (week 70 64.7 pmol/L [121.5]; LOV 76.8 [140.2] pmol/L; baseline 20.2 pmol/L [9.5]; normal range 1.8–9.2 pmol/L; Fig. 5a). As reported previously, mean (SD) levels of adrenal hormone precursors, testosterone and renin increased from baseline to the end of the core phase (week 22) [15]. Mean levels of adrenal hormone precursors (11-deoxycortisol and 11-deoxycorticosterone) decreased during long-term osilodrostat treatment (11-deoxycortisol: week 70 20.7 nmol/L [17.7]; LOV 14.1 nmol/L [16.4]; baseline 4.5 nmol/L [4.9]; normal range 0–3.92 nmol/L; 11-deoxycorticosterone: week 70 2674.8 pmol/L [2064.8]; LOV 2225.8 pmol/L [1824.9]; baseline 266.6 pmol/L [325.0]; normal range 0.05–0.39 nmol/L; Fig. 5b and c). In females, mean (SD) testosterone levels decreased towards baseline values during the extension phase to within the normal range at LOV (week 70 1.9 nmol/L [1.4]; LOV 1.6 nmol/L [1.4]; baseline 1.2 nmol/L [0.7]; normal range 0.1–1.6 nmol/L; Fig. 5d). No new or worsening cases of hirsutism were reported during the extension phase. In males, testosterone levels increased to week 70 and stabilized thereafter; levels remained within the normal range during the extension (week 70 21.0 nmol/L [12.6]; LOV 14.0 nmol/L [9.4]; baseline 7.4 nmol/L [3.5]; normal range 8.7–38.2 nmol/L; Supplementary Fig. 3a). Mean (SD) renin levels increased up to week 70 and stabilized thereafter (week 70 61.0 mU/L [98.2]; LOV 42.1 mU/L [47.4]; baseline 34.9 mU/L [50.6]; normal range not available; Supplementary Fig. 3b). In the core study, mean (SD) aldosterone levels decreased from baseline to week 22. During the extension, aldosterone levels increased up to week 70 and stabilized thereafter, with levels remaining within the normal range for the duration of the study (week 70 10.9 pmol/L [15.4]; LOV 34.6 pmol/L [49.9]; baseline 156.9 pmol/L [235.7]; normal range 55–250 pmol/L; Supplementary Fig. 3c). During the core phase, estradiol levels in males decreased from baseline to week 22 and decreased and stabilized during the extension (week 70 78.1 pmol/L [73.0]; LOV 54.8 pmol/L [27.0]; baseline 59.4 pmol/L [24.4]; normal range not available; Supplementary Fig. 3d).

### Safety

All 19 enrolled patients experienced at least one AE during the study. The most common AEs, regardless of study drug relationship, reported during the core and extension phases are listed in Tables 2 and 3. Overall, three patients discontinued the study because of AEs.

**Table 2:** AEs (≥ 2 patients) regardless of study drug relationship reported during the core phase (n = 19)

	All grades n	Grade ≥ 3 n
AEs regardless of study drug relationship		
Blood corticotropin increased	7	0
Hormone level abnormal	7	0
Asthenia	6	0
Nausea	6	0
Fatigue	4	0
Headache	4	0
Nasopharyngitis	4	0
Acne	3	0
Blood creatinine phosphokinase increased	3	1
Blood testosterone increased	3	0
Diarrhea	3	0
Dizziness	3	0
Hypertrichosis	3	0
Malaise	3	0
Adrenal insufficiency	2	1
Anemia	2	1
Back pain	2	0
Depression	2	1
Hirsutism	2	0
Hypomagnesemia	2	2
Muscle spasms	2	1
Musculoskeletal stiffness	2	0
Pruritus	2	0
Rash	2	0
Toothache	2	0
Sinus bradycardia	2	0
Urinary tract infection	2	0
Vitamin D decreased	2	0

A patient with multiple severity grades for an AE is only counted under the maximum grade

**Table 3:** AEs (≥ 2 patients) regardless of study drug relationship reported during the extension phase (n = 16) and AEs of special interest reported during the entire study (n = 19)

	All grades n	Grade ≥ 3 n
AEs regardless of study drug relationship		
Adrenal insufficiency	7	1
Arthralgia	6	0
Headache	6	1
Diarrhea	5	0
Abdominal pain	4	1
Urinary tract infection	4	0
Arthropod bite	3	0
Blood testosterone increased	3	0
Edema peripheral	3	0
Fatigue	3	0
Hypertension	3	3
Lipase increased	3	1
Vertigo	3	0
Asthenia	2	0
Blood corticotropin increased	2	0
Blood pressure increased	2	0
Dizziness	2	0
Gastroenteritis	2	1
Hypokalemia	2	0
Influenza	2	0
Insomnia	2	0
Iron deficiency anemia	2	0
Musculoskeletal pain	2	0
Nasopharyngitis	2	0
Non-cardiac chest pain	2	1
Pain in extremity	2	0
Pituitary-dependent Cushing’s syndrome	2	2
Upper respiratory tract infection	2	0
Vomiting	2	0
Vulvovaginal mycotic infection	2	0
Weight decreased	2	1
Weight increased	2	0
AEs of special interest		
Related to accumulation of adrenal hormone precursors	12	4
Related to hypocortisolism	11	2
Related to arrhythmogenic potential	1	0
Related to QT prolongation	1	0

A patient with multiple severity grades for an AE is only counted under the maximum grade. Pituitary-dependent Cushing’s syndrome was the preferred term used by investigators to report events of worsening or uncontrolled Cushing’s syndrome

Of all patients who experienced at least one AE, 18 patients experienced an AE suspected to be related to osilodrostat treatment (Supplementary Table 1). The most common AEs reported during the entire study period that were suspected to be related to osilodrostat treatment were adrenal insufficiency (n = 9), increased blood corticotropin (n = 8), nausea (n = 7) and abnormal hormone levels (n = 7). Most AEs were grade 1 or 2 in severity. The most commonly reported grade ≥ 3 AEs were hypertension (n = 4), pituitary-dependent Cushing’s syndrome (n = 3) and adrenal insufficiency (n = 2). Three of the four cases of hypertension (grade ≥ 3) were reported during the extension phase. Of five patients who experienced an AE of hypertension and/or increased blood pressure, three had high SBP and/or DBP at baseline (SBP > 130 mmHg and DBP > 90 mmHg at baseline, n = 1 [SBP 149.3 mmHg; DBP 101.3 mmHg]; SBP > 130 mmHg at baseline, n = 1 [169.7 mmHg]; DBP > 90 mmHg at baseline, n = 1 [91.7 mmHg]). At LOV, two patients had SBP > 130 mmHg and DBP > 90 mmHg (SBP 149 and 160 mmHg; DBP 96 and 108 mmHg, respectively). One patient had SBP > 130 mmHg (150.3 mmHg), and two patients had DBP > 90 mmHg (96.3 and 91.7 mmHg).

Overall, eight patients experienced an AE of increased ACTH for a mean (SD) duration of 87.7 weeks (121.5). All cases were grade 1 or 2 in severity. Of eight patients who experienced an AE of increased ACTH, six also experienced an AE related to hypocortisolism. These AEs mostly occurred during the extension phase and were mostly grade 1 or 2 in severity; grade 3 AEs of increased blood corticotropin were reported in two patients.

Throughout the entire study, when AE terms were grouped into AEs of special interest, 12 patients experienced an AE relating to accumulation of adrenal hormone precursors. Such AEs included acne (n = 2), hirsutism (n = 2), hypertension (n = 2), hypertrichosis (n = 2), hypokalemia (n = 2), blood potassium decreased (n = 1) and weight increased (n = 1). A patient was only counted once if they experienced more than one AE in this category. These AEs were managed with dose adjustment and additional therapy in two and 10 patients, respectively.

Hypocortisolism-related AEs were reported in 11 patients and were managed with dose interruption in each of these patients and with additional therapy in four patients. No patients permanently discontinued osilodrostat treatment because of AEs relating to accumulation of adrenal hormone precursors or hypocortisolism. Only one patient reported an AE related to arrhythmogenic potential (syncope); this AE occurred during the extension and was managed with dose adjustment. An AE related to QT prolongation (QTcF prolongation) was reported in only one patient during the core phase, as reported previously [15]; the patient was hospitalized and managed with adjustment of osilodrostat dose (Table 3). No new AEs relating to QT prolongation were reported during the extension. Two patients discontinued during the extension phase because of AEs (blood corticotropin increased and neoplasm progression, n = 1; benign pituitary tumor, n = 1).

Analysis of change in pituitary tumor volume during the study was not possible as only two patients had an evaluable magnetic resonance imaging assessment at baseline and a post-baseline visit.

## Discussion

To our knowledge, LINC 2 is the longest prospective study to date of a steroidogenesis inhibitor in patients with Cushing’s disease. The final data reported here show that osilodrostat provides sustained reductions in mUFC levels in patients with Cushing’s disease for up to 6.7 years of treatment. Reductions in mUFC levels were accompanied by rapid and sustained reductions in serum and salivary cortisol levels, as well as continued control of most cardiovascular-related parameters. No new safety signals were reported.

Normalization of cortisol levels is an important goal in the management of Cushing’s disease [1, 4]. In this study, osilodrostat led to a rapid, robust and durable reduction in mean mUFC from a baseline value of ~ 10 × ULN to within the normal range. This rapid reduction in cortisol levels is important to alleviate the clinical burden of disease and improve QoL in patients with Cushing’s disease. This supports the findings of two prospective, Phase III studies of osilodrostat in which rapid reductions in cortisol levels were also observed [16, 17]. The proportion of patients with a complete (mUFC ≤ ULN) or partial (mUFC > ULN but ≥ 50% reduction from baseline) mUFC response remained ≥ 50% at all time points during the extension period up to month 70. Few (n ≤ 6) patients had evaluable assessments at time points after month 70, which limits interpretation of response rate after this time point. While the proportion of patients with mUFC ≤ ULN was higher at the start of the extension phase (week 22; 78.9%) than at subsequent time points up to month 70 (50.0%), it is important to note that patients who discontinued the study were classified as non-responders up to the last visit they could have completed. Furthermore, these findings could not have been influenced by prior pituitary irradiation: only one patient underwent gamma radiation therapy (more than 5 years prior to the start of the study). Indeed, only two patients met the study criteria for loss of mUFC response (mUFC > ULN on ≥ 2 consecutive visits at the highest tolerated dose of osilodrostat after previous mUFC normalization); mUFC levels in both patients increased to > ULN following a reduction in osilodrostat dose for an AE. The long-term benefit of osilodrostat is further demonstrated by the observation that over half of all enrolled patients received over 4.5 years of osilodrostat treatment (median drug exposure 5.4 years).

Cardiovascular disease is one of the leading causes of death and excess morbidity among patients with Cushing’s disease [2, 18]. Common cardiovascular and metabolic conditions associated with hypercortisolism include hypertension, impaired glucose tolerance, dyslipidemia and obesity [19]. In this study, most cardiovascular-related parameters (including control of glycemic parameters, blood pressure, weight and BMI) were controlled during long-term osilodrostat treatment. There were few clinically relevant changes in cardiovascular-related parameters noted in the core phase; this may be because of the short duration of the core phase (22 weeks) and the lack of time to observe any related changes in these parameters. FPG, HbA_1c_, SBP, DBP, total cholesterol, LDL cholesterol, HDL cholesterol and triglycerides were close to or within the normal range at baseline, which could explain the lack of reductions observed in some patients during the study. However, the data show that control of these parameters was maintained during long-term osilodrostat treatment. These findings may be clinically relevant given that Cushing’s disease is a progressive disorder and sustained control of cardiovascular-related parameters alleviates the clinical burden of Cushing’s disease and improves QoL. There was high variability in mean change from baseline in some lipid parameters, which may have also contributed to the lack of clear changes in lipid parameters observed during long-term osilodrostat treatment. However, in a large, pivotal, 48-week Phase III study of osilodrostat (LINC 3), improvements in lipid parameters (total and LDL cholesterol) were observed from baseline to the end of the core study (week 48) [16]. The clinical benefits observed in this analysis are consistent with data from LINC 4, which showed that improvements in most cardiovascular-related parameters from baseline were first observed during the initial 12-week dose-titration period and were maintained up to the end of the core study (week 48) [17].

Osilodrostat was generally well tolerated during long-term treatment; the overall safety profile for up to 6.7 years of treatment (median duration of treatment 5.4 years) was consistent with that reported during the core phase of the study [15]. Most AEs were of mild or moderate severity, and few patients needed to discontinue treatment for AEs (3/19; 15.8%), with no patients discontinuing the study because of AEs relating to hypocortisolism or accumulation of adrenal hormone precursors. AEs potentially related to hypocortisolism were reported in 57.9% of patients. Hypocortisolism-related AEs included the following terms: adrenal insufficiency, cortisol decreased, cortisol free urine decreased, and glucocorticoid deficiency. These AEs were manageable with dose interruption and/or adjustment, without the need for permanent discontinuation of osilodrostat, consistent with other findings [16].

Accumulation of adrenal hormone precursors is expected based on the mechanism of action of osilodrostat [11]. AEs relating to accumulation of adrenal hormone precursors occurred in 63.2% of patients, were mostly mild to moderate in severity, and were managed with dose interruption and/or additional therapy. Interestingly, initial increases in mean levels of adrenal hormone precursors (11-deoxycortisol and 11-deoxycorticosterone) during osilodrostat treatment were followed by decreases towards baseline values during the extension phase. Given the potential risk of adrenal insufficiency with any cortisol-lowering drug and the, albeit infrequent, observation of a newly occurring AE of hypertension during the extension phase, it is important to monitor blood pressure during osilodrostat treatment.

Excess production of testosterone in females treated with osilodrostat has been previously described [10]. Mean levels of testosterone in females in this study followed a similar pattern to those of adrenal hormone precursors and decreased towards baseline levels during long-term treatment after an initial increase during the first 22 weeks of the study; testosterone levels in females were within the normal range at LOV. In line with the reduction in testosterone after week 22, no new or worsening cases of hirsutism were reported during the extension phase. Testosterone levels in male patients remained within the normal range for the duration of the study. These findings are consistent with those reported in the long-term extension to the prospective, Phase III, LINC 3 study, where testosterone levels decreased towards baseline levels during the extension following an initial increase during the core phase [20].

These data are limited by the small number of patients who were enrolled in this open-label study. Nonetheless, they provide valuable insights into long-term osilodrostat treatment in patients with Cushing’s disease, with continued treatment for up to 6.7 years. In addition, changes in pituitary tumor volume could not be assessed during this study as only two patients had a measurable tumor volume at both baseline and during the extension phase; therefore, correlations between pituitary tumor volume and ACTH could not be determined. However, ACTH levels remained stable over long-term treatment.

## Conclusions

The efficacy and tolerability data reported from this Phase II long-term study show that osilodrostat provides sustained control of cortisol levels and improvements in cardiovascular-related parameters for up to 6.7 years of treatment. Notably, following an initial increase during the core phase, testosterone levels decreased towards baseline values in females during long-term treatment. These data support the use of long-term osilodrostat as an effective treatment option in patients with persistent, recurrent or de novo Cushing’s disease.

## Supplementary Information

Below is the link to the electronic supplementary material.

Supplementary file1 (DOCX 260 kb)

## Data Availability

The datasets generated and analyzed during the current study are not publicly available but are available from the corresponding author on reasonable request. Recordati Rare Diseases will share the complete de-identified patient dataset, study protocol, statistical analysis plan, and informed consent form upon request, effective immediately following publication, with no end date.
