# Supplementary material for: Long-term efficacy and safety of osilodrostat in Cushing’s disease: final results from a Phase II study with an optional extension phase (LINC 2)
Source: Pituitary. 2022 Oct 11;25(6):959–70. doi: 10.1007/s11102-022-01280-6 (PMC9675663; doi:10.1007/s11102-022-01280-6)
Supplement: Supplementary file 1 — Supplementary file1 (DOCX 260 kb) [file 11102_2022_1280_MOESM1_ESM.docx]

**Long-term efficacy and safety of osilodrostat in Cushing’s disease: final results from a Phase II study with an optional extension phase (LINC 2)**

**Supplementary material**

**Supplementary Figure 1. Individual patient data for (a) morning serum cortisol, (b) morning salivary cortisol and (c) late-night salivary cortisol levels**


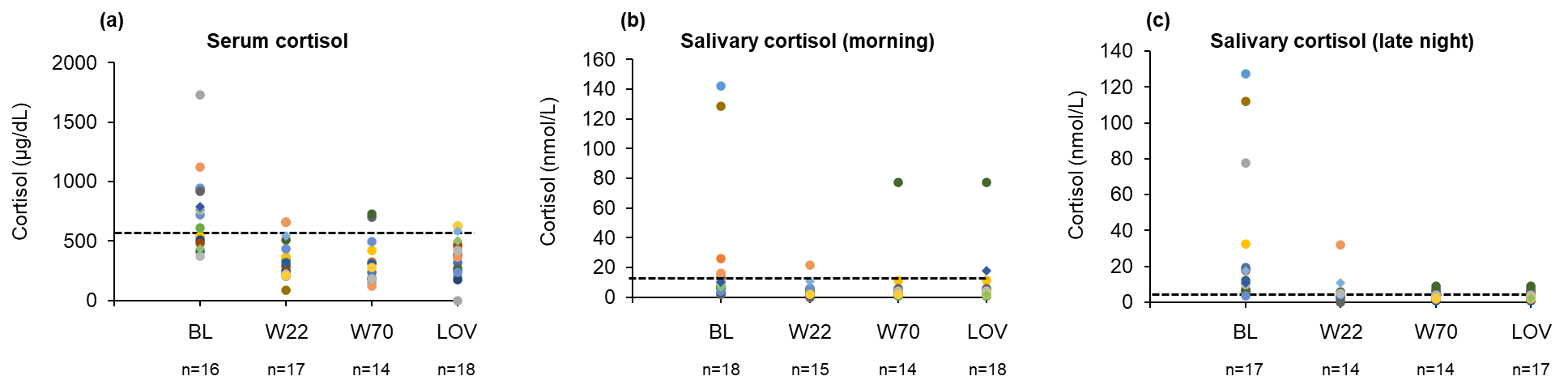


Dashed lines represent the ULN: morning serum cortisol 567 nmol/L; morning salivary cortisol,
15.5 nmol/L; late-night salivary cortisol, ≤2.5 nmol/L. Shaded areas highlight data that have been published previously [1]. BL, baseline; LOV, last observed value; ULN, upper limit of normal; W, week

**Supplementary Figure 2. Individual patient data for (a) total cholesterol, (b) HDL cholesterol, (c) LDL cholesterol and (d) triglycerides**

**
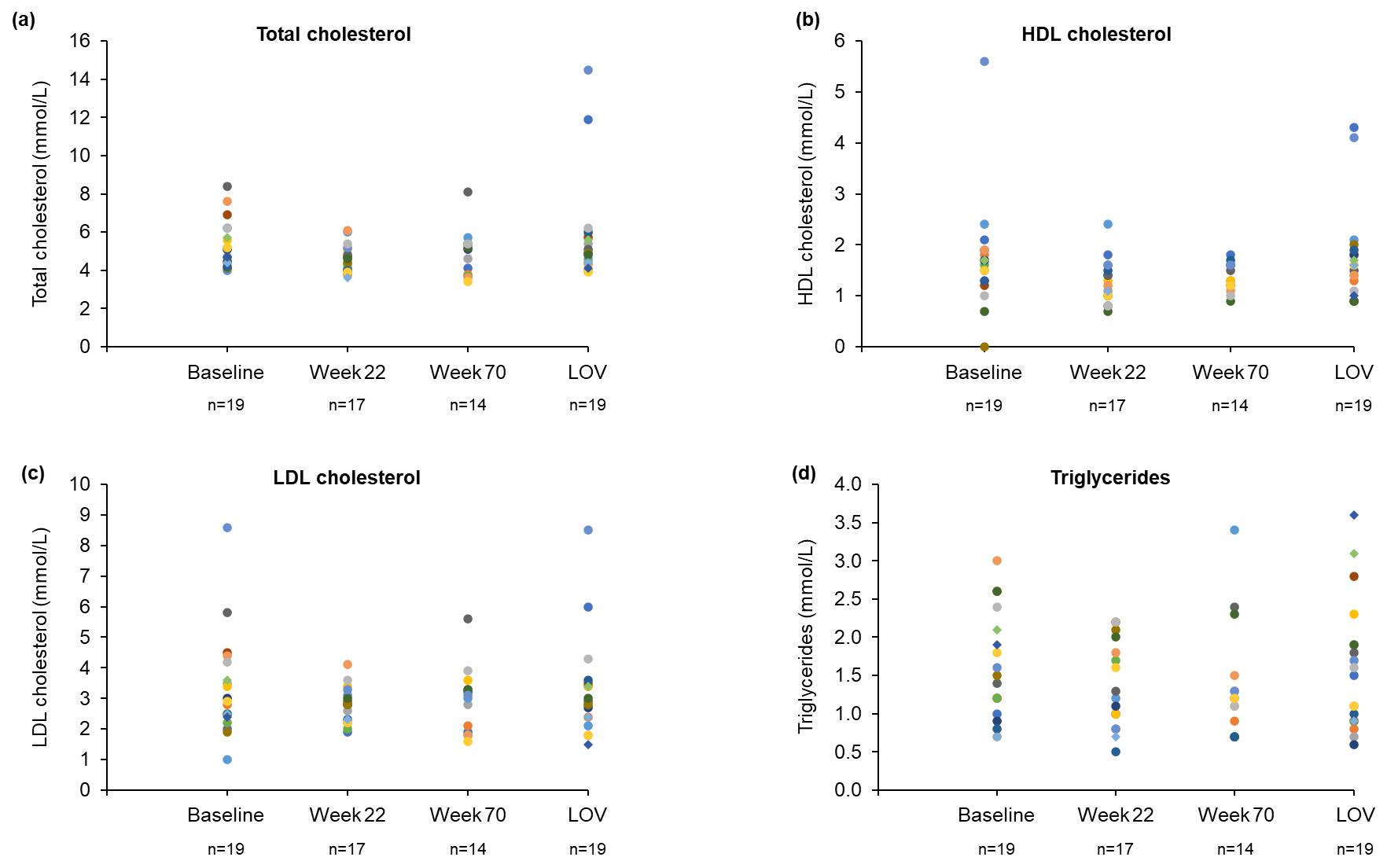
**

HDL, high-density lipoprotein; LDL, low-density lipoprotein

**Supplementary Figure 3. Individual patient data for (a) testosterone (males), (b) renin, (c) aldosterone and (d) estradiol (males) levels**

**
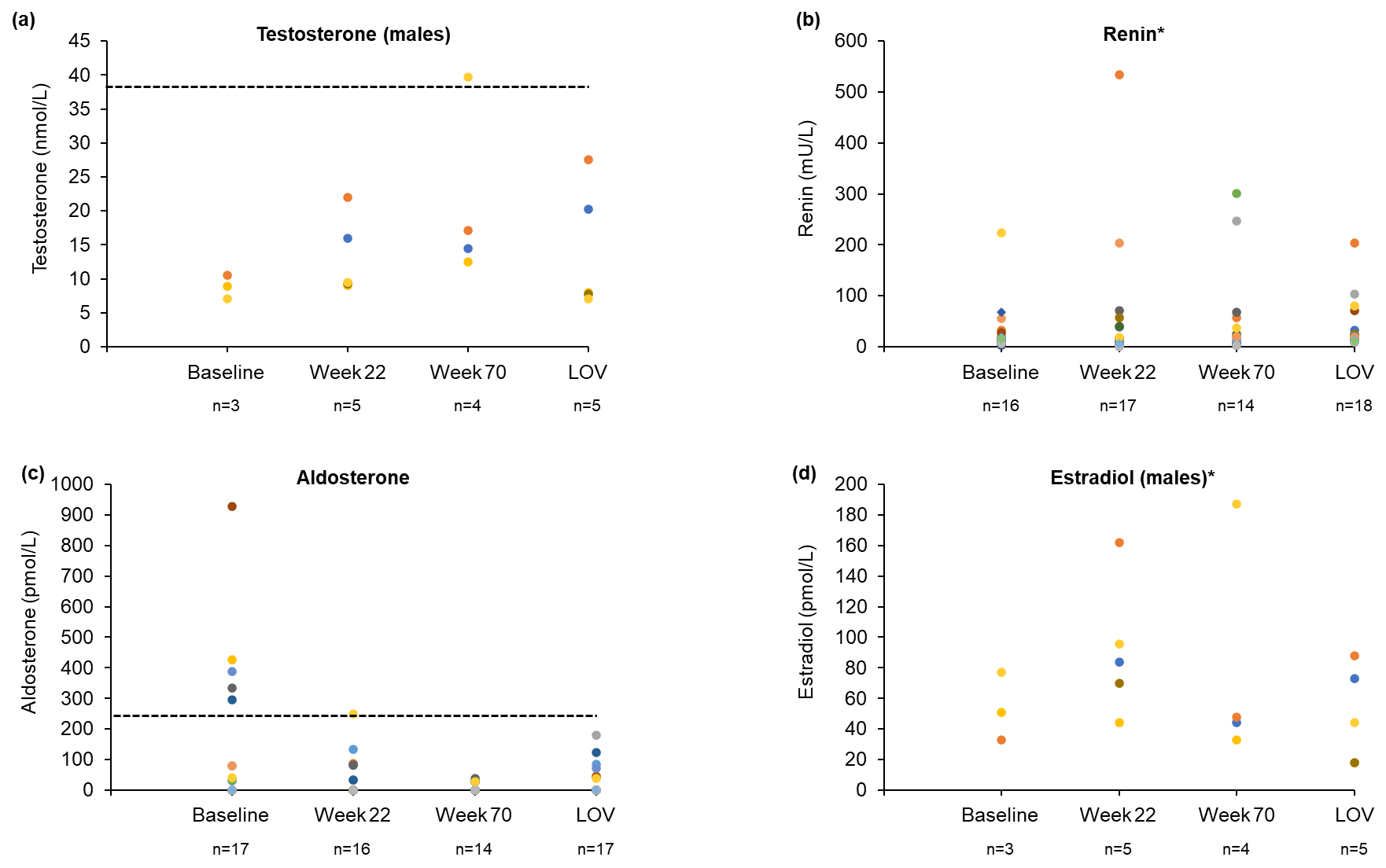
**

Dashed lines represent the ULN: testosterone (males), 38.2 nmol/L; aldosterone, 250 nmol/L. *ULN not available

**Supplementary Table 1. AEs suspected to be related to osilodrostat treatment (≥10% of patients) (n=19)**

|  | **All grades n (%)** | **Grade ≥3 n (%)** |
| --- | --- | --- |
| **AEs suspected to be related to study treatment** |  |  |
| Adrenal insufficiency | 9 | 2 |
| Blood corticotropin increased | 8 | 0 |
| Hormone level abnormal | 7 | 0 |
| Nausea | 7 | 0 |
| Blood testosterone increased | 6 | 0 |
| Fatigue | 5 | 0 |
| Asthenia | 4 | 0 |
| Diarrhea | 3 | 0 |
| Acne | 2 | 0 |
| Headache | 2 | 0 |
| Hirsutism | 2 | 0 |
| Hypertension | 2 | 2 |
| Hypertrichosis | 2 | 0 |
| Hypokalemia | 2 | 0 |
| Sinus bradycardia | 2 | 0 |
| Weight decreased | 2 | 1 |

AE, adverse event
